# Women’s lived experience of endometriosis-related fertility issues

**DOI:** 10.1371/journal.pone.0293531

**Published:** 2023-11-06

**Authors:** Elodie Girard, Anna Mazloum, Isabelle Navarria-Forney, Nicola Pluchino, Isabelle Streuli, Christine Cedraschi

**Affiliations:** 1 Division of Liaison Psychiatry and Crisis Intervention, Department of Psychiatry, Geneva University Hospitals, Geneva, Switzerland; 2 Private Practice, Centre Médical de Lancy, Geneva, Switzerland; 3 Division of Gynaecology, Lausanne University Hospitals and the Faculty of Medicine of Lausanne, Lausanne, Switzerland; 4 Division of Gynaecology, Reproductive Medicine Unit, Geneva University Hospitals and the Faculty of Medicine of University of Geneva, Geneva, Switzerland; 5 Division of General Medical Rehabilitation, Department of Geriatrics and Rehabilitation, Faculty of Medicine, Geneva University Hospitals (HUG), University of Geneva, Geneva, Switzerland; University of Melbourne, AUSTRALIA

## Abstract

**Objective:**

The aim of the present study is to conduct a qualitative investigation to provide a deeper understanding of women’s views about endometriosis, fertility and their perception of reproductive options.

**Methods:**

Semi-structured interviews were conducted by two female psychiatrists, specialized in gynecology and obstetrical consultation-liaison psychiatry, trained in qualitative procedures, with experience in qualitative studies and in psychological support of women attending infertility consultations. No prior relationship with respondents was established before data collection. Interviews were tape-recorded and transcribed. Interviews lasted 45–75 minutes. The transcripts were then analysed using thematic content analysis.

**Results:**

Twenty-nine women were contacted. Twelve agreed to an interview at the hospital’s infertility clinic. Eleven women with diverse sociodemographic characteristics were included. The key findings of thematic content analysis can be grouped into four topics: (1) Diagnostic announcement and initial delay; (2) Negative perceptions of initial care: pre-diagnosis phase; (3) Struggle with endometriosis and its treatment; (4) Issues related to health problems, fertility and reproductive options.

**Conclusion:**

Our analysis of the interviews corroborates the distressing impact of the trivialization of pain and the uncertainty of or the long quest for diagnosis. The findings also stress various associated issues, from the diagnostic delay to the low success rates of fertility treatments. This qualitative analysis contributes to better understand the accumulation of negative emotions within the illness trajectory and the poor dyadic adjustment within the couple.

## Introduction

Endometriosis is an enigmatic, chronic, and recurring disease affecting up to 10% of reproductive-age women worldwide [1]. It is associated with a significant burden on the woman, her family, and society. Endometriosis has profound effects on women’s lives.

Many facets of endometriosis, including pathogenesis and treatment, are not fully elucidated [2]. Most common symptoms include dysmenorrhea, non-cyclic pelvic pain, dyspareunia and dyschezia [1]. There is little correlation between the stage and extension of endometriosis lesions and symptom severity [3]. There is, currently, no cure for endometriosis and treatments focus on symptom management. They include hormonal therapy and surgery, often associated with a poor outcome in terms of satisfaction and long-term relief [4]. Endometriosis is associated with infertility and quoted statistics are that 30–40% of women with endometriosis experience infertility [5, 6].

While the relationship between endometriosis and infertility remains unclear, there is strong evidence that potential or diagnosed infertility can cause a considerable emotional [7] and financial [8–10] burden. Previous research on women’s experiences of infertility suggest that women benefit from supportive care and timely treatment [11, 12]. However, little is known about understanding of the effects of endometriosis on women’s lives and facing this complex condition. In particular, the women’s views regarding symptom onset and the further course of the disease and its impact on fertility has not been explored.

Women with endometriosis are confronted with various possibilities and choices related to family planning and fertility, such as elective oocytes vitrification, surgical removal of endometriomas, assisted reproductive techniques (ART), or oocyte donation or adoption. In 2018, Navarria-Forney et al. conducted a quantitative web-based survey on fertility issues in women aged 18–40 years with endometriosis. Issues such as the wish of having children, knowledge and attitudes toward endometriosis and fertility, means used to access information, and reproductive choices were addressed [13]. Findings showed that almost all women (96%) worried about the impact of endometriosis on their fertility. Approximately half of them (52%) reported having received sufficient information concerning the effect of endometriosis on fertility from their doctor, whereas 31% had discussed fertility issues with their doctor but desired further information. In contrast, only a minority (27%) of women considered themselves well-informed on fertility preservation options. Information given by specialists on endometriosis and reproduction was considered most useful. Information mediated through patient support groups was also highly rated, whereas information given by the general gynecologist was less highly rated. Most women would consider ART (74%) or adoption (70%) in case of infertility. Interestingly, 72% of women would undergo oocyte vitrification for fertility preservation, whereas only 37% would resort to oocyte donation. The need for a qualitative analysis exploring illness perceptions emerged when designing the research protocol.

Research on illness perceptions conceptualizes how individuals form cognitive and emotional perceptions after experiencing health threats [14]. The illness perception model refers to the importance of subjective, organized beliefs about the symptoms, consequences, time course, controllability, and course of an illness. Illness perceptions have been shown to predict a range of psychosocial and clinical outcomes such as coping strategies and psychological adjustment to medical conditions [15–18]. Infertility raises issues related to one’s own finitude and choices [19]. Inability to conceive represents an existential crisis in the sense of questioning one’s own physical and psychological integrity [20, 21]. The desire for a child and the investment in the process from conception to pregnancy are at the center of a cluster of mental representations that touch on intimate and essential questions such as parenthood, filiation, sexuality, belonging to a community, death and love [22, 23]. Research investigating the role of illness perceptions in medical conditions has developed extensively in recent years, with a special focus on understanding how patients’ perceptions influence adherence, treatment decisions, risk perception and health behaviors as well [16, 24, 25].

The aim of the present study is to further elaborate on the quantitative study by a qualitative investigation to provide a deeper understanding of the women’s views about endometriosis, fertility and their perception of reproductive options.

## Methods

All subjects were informed of the goals and design of the study and assured of confidentiality before formally agreeing to participate. Written informed consent was obtained from all participants. Data were rendered anonymous after data collection to ensure confidentiality. Participants were recruited between January and December 2019.

### Study design

Qualitative research methods (i.e., a grounded theory, open-enquiry approach) were used to investigate participants’ perceptions and descriptions of their endometriosis and concerns about fertility issues.

Semi-structured interviews were conducted by two female psychiatrists, specialized in gynecology and obstetrical consultation-liaison psychiatry, trained in qualitative procedures, with experience in qualitative studies and in psychological support of women attending infertility consultations. No prior relationship with respondents was established before data collection.

### Procedure

Eleven participants who had responded to the online quantitative survey [13] and who had accepted to be contacted for a qualitative interview were recruited. These participants were questioned using face-to-face semi-structured interviews, conducted in French to elicit patients’ views on what triggered their endometriosis, on the possible causal explanations of the problem and on the associations, they make with infertility.

An interview guide was developed including general topics about symptom onset (Table 1). The items were not addressed in a fixed order, although the first question was always “Tell me about your endometriosis, how did it start?” The interview also investigated their views on the circumstances surrounding the onset and the development of pain and endometriosis-related issues. As the interview progressed, issues about the causes and consequences of the symptoms related to the diagnosis were addressed, as well as the treatment, the relationships with the gynecologist or specialists in fertility and other care providers. The aim was to access a large range of perceptions about endometriosis, fertility as well as the women’s perceptions of the health care system. Interviews were tape-recorded and transcribed verbatim and edited to erase all possible identifying information The interview guide was pilot tested with two patients. Interviews lasted 45–75 minutes. The transcripts were then analysed using a thematic content analysis method [26, 27].

**Table 1:** Interview guide and topics.

Queries	Topics
Tell me about your endometriosis, how did it start?	Illness onset
When was it diagnosed? How long did you experience symptoms before the diagnosis was made?	Time to diagnosis
What type of treatment were you given?	Treatment
What do imagine as a cause for your symptoms? What are the consequences of your symptoms in your daily life?	Causes and consequences of the symptoms related to the diagnosis
How would you describe your relationships with your therapists? And with your family and your partner (if applicable)?	Issues related to relationships (patient-therapist, partner, others)
How did you figure out possible consequences on fertility?	Issues related to fertility

### Data analysis

The qualitative analysis began with close readings of the transcripts by two researchers working separately. The analysis continued throughout data collection and coding process. Based on these transcripts, thematic content analysis led to the identification of categories and themes using the constant comparative method that consists of analyzing the interviews by comparing one response with earlier observed responses [27, 28]. The readings were compared and subsequently used to establish analytical categories. These categories served as a basis for the final grid, which was then used independently by the two researchers to analyze the transcripts to maximize theoretical sensitivity and rigor [29]. A third senior investigator joined the multidisciplinary research team (psychologist, psychiatrists, and gynecologists) to discuss and refine the final grid which was then used to analyze the transcripts. Finally, background data were provided to ascertain the context of the study and to allow comparisons to be made.

Using patient-generated data via the interviews and verification of interpretation using the research team allowed for an assessment of trustworthiness [30]. Familiarity with the culture and adequate understanding of the population of patients has been developed before the first data collection. A triangulation was used insofar as two investigators collected and analyzed the raw data so that findings emerged from consensus within the multidisciplinary team.

The sample of participants investigated allowed to reach a point at which no new categories emerged from transcript analysis [31, 32]. Thus, data saturation, defined as the point in data collection and analysis at which new information produces little or no change in the codebook was achieved [33, 34].

## Results

### Participants

Twenty-nine women were contacted. Twelve agreed to come to the HUG Infertility center for the interview. Eleven women were included, with diverse sociodemographic characteristics (Table 2). Only one was excluded because of no desire of motherhood and no discussion about infertility (n = 10).

**Table 2:** Respondents’ characteristics.

	Age	Profession	Marital status
Endo 1	19 years	Student	Living with boyfriend
Endo 2	39 years	Secretary	Married
Endo 3	28 years	Civil servant	Single
Endo 4	33 years	Teacher	Living with boyfriend
Endo 5	25 years	Saleswoman	Married
Endo 7	38 years	Project manager	Married
Endo 8	32 years	Nurse	Living with boyfriend
Endo 9	32 years	Instructor	Single
Endo 10	34 years	Complementary medicine therapist	Single
Endo 11	34 years	Project manager	Married

### Perceptions of women’s experience of endometriosis

**Fig 1:**
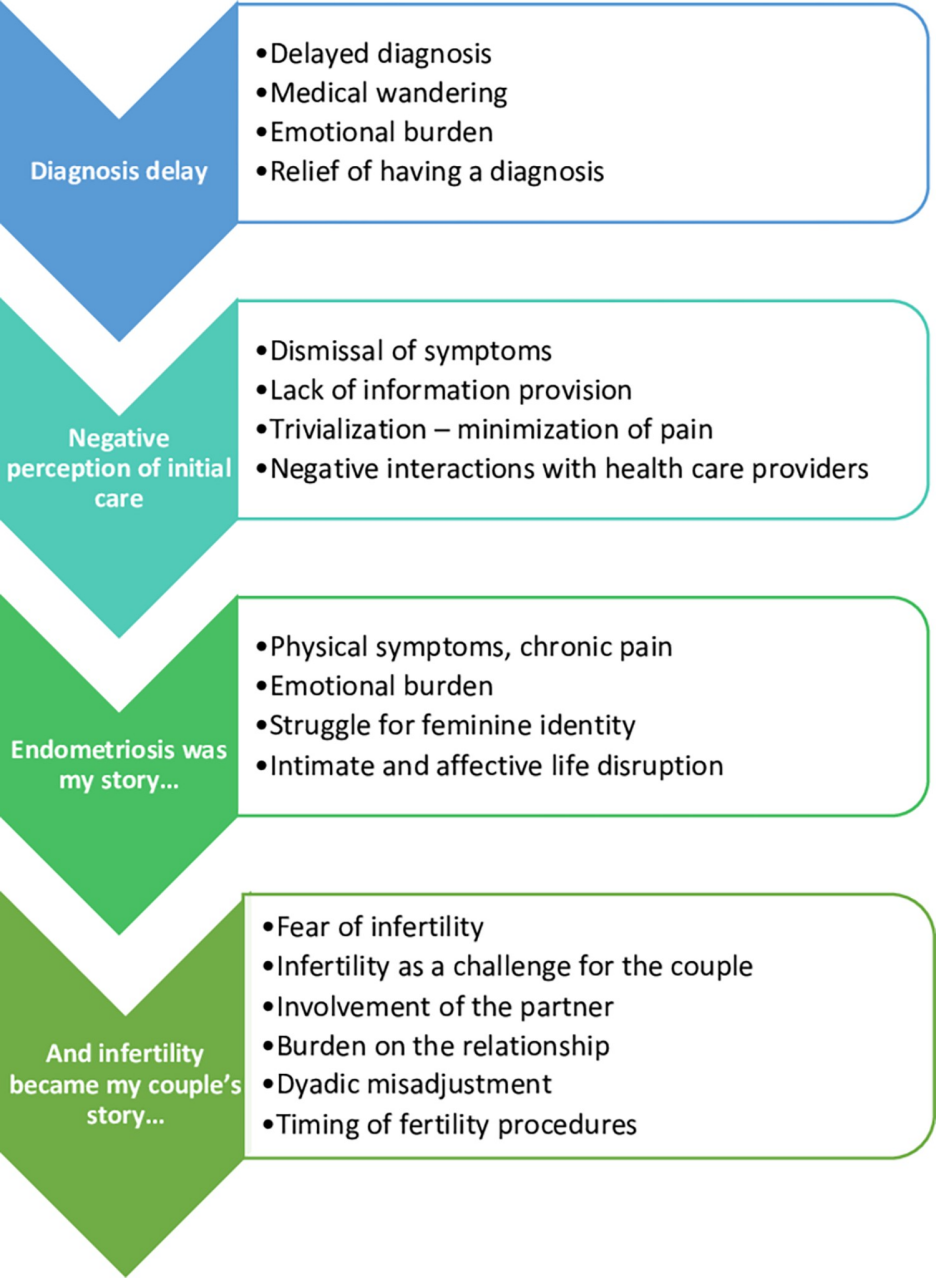
Summary of emerging themes.

Throughout the interviews, participants shared their experiences and difficulties regarding endometriosis and fertility. It is key to note that our respondents, whatever their age, were ready to discuss fertility issues and ART options but, after and only after, they had expressed in depth the emotional and physical burden of endometriosis within their life trajectories. The key findings of thematic content analysis can be grouped into the following four topics: (1) Diagnostic announcement and initial delay; (2) Negative perceptions of initial care: phase of pre-diagnosis; (3) Struggle with endometriosis and its treatment; (4) Issues related to health problems, fertility and reproductive options (Fig 1).

#### 1. Diagnostic delay

*Difficult menarche and dysmenorrhea*. When asked about the beginning of the symptoms of endometriosis, nine out of eleven women were able to precisely date it. For most of them, it started during their adolescence or young adulthood. Three of them relate an abrupt start at menarche: one remembers *“the first day of my period was horrible*. *I had pain in the lower abdomen and was doubled over pain*. *It really*, *really hurt” (Respondent 9)*. Only one woman experienced the first symptoms in her thirties.

Seven of the women who had a delayed diagnosis claimed the implication of this delay on the unfavorable evolution of the disease. “*It was a pretty long medical wandering concerning my disease*. *When I was 13*, *I was moving from one pill to another to calm the symptoms*, *but never focusing on the real problem and the disease*. *When I started considering having kids*, *doctors took it seriously*, *but the extent of the damage inside was advanced* “(Respondent 5).

Women’s narratives frequently attested to a lack of true acknowledgment regarding their menstrual pain, thus conducting to a long medical wandering before a diagnosis was considered.

“*It is a complex condition hard to detect…I think it has started a long time ago now; it’s not evident because during periods*, *we women have pain and we grow up with this idea that it’s normal and therefore we don’t worry that much…I was even telling myself*, *I’m stressed…*, *I’m somatizing pain in my belly… but later one day*, *after having had sex*, *it finished with a bloodbath and a piece of flesh*, *and we went to the emergency at the hospital*” (Respondent 4). Underlying the dramatic issue of the lived experience of intimacy and sexuality, this narrative is also showing the emotional burden related to the condition and the traumatic context in which the diagnosis has become obvious in this case.

Three out of ten participants said that the diagnosis of endometriosis was relatively quickly mentioned and that they were rapidly referred to a specialist. Two of them were firstly mistreated for ovarian cyst and the surgery led to an early diagnosis of endometriosis.

Most of the women reported that they have developed a strong pain tolerance. They said things like “*I always had painful periods*, *it’s normal […] all the women in my family have painful periods*” (Respondent 7). “*I was 23 when I was diagnosed with endometriosis*. *It was due to severe pain during menstruation*. *Pain that prevented me from doing anything*. *I was bedridden*, *stabbed in the stomach and thought to myself that maybe this is normal*. *My sister was in pain too*, *so I thought maybe it wasn’t that alarming*. *It’s normal to have pain during your periods*.” (Respondent 11)

The role of the gynecologist or general practitioner in the detection of the pain symptoms in this population of teenagers or young women appears to be a central issue to be taken into consideration since the initial diagnosis is known to have an impact on psychological coping as well as on the illness-related perception.

#### 2. Negative perceptions of initial care: Phase of pre-diagnosis

Most of the women describe how their primary health care provider responded to their complaints. Nine out of eleven women said that gynecologist did not acknowledge their complaints. One complained of the lack of information, saying that the diagnosis of endometriosis was mentioned but not explained, and minimized. Another complained that her gynecologist was not familiar with endometriosis and that therefore the medical anamnesis was incomplete. The same feeling was reported from the hospital emergency consultation: “*Very often*, *we have the impression of being “sissy” or “imaginary sick”*. *We went back and forth to the Emergency Room during the cycles*. *I really was in pain*. *I was told that it was nothing*, *just exam stress*. *But in the end*, *there really was a problem*.*” (Respondent 5)*.

Yet, as this respondent also indicated “*Once you have found the right professionals who are interested in the disease*, *you finally feel heard*. *But before that*, *we walked from one doctor to the other*. *You feel like a guinea pig*. *Sometimes you feel like you know more because you are trying to learn*, *to know more about the disease*. *It’s quite tricky to have these professionals in front of us who don’t answer clearly”*.

Such narratives are underlining the relieving effect of identifying a diagnosis and its consecutive reassuring value in terms of clarification, regaining control and secondary better adjustment to the condition: “*I get along very well with my specialist*, *he was always very clear when I had questions and always answered*. *We were able to discuss*, *and he explained to me very well so I never felt overwhelmed after*” (Respondent 4). In fact, patients’ experiences with specialists appear to be positively related to their attentive listening, provision of information and answers to the patient’s questions about diagnosis and treatment.

On the contrary, the trivialization and minimization of the pain, the uncertainty or absence of diagnosis and the long struggle related to the medical wandering represent negative perceptions which are known to hinder positive adjustment to chronic pain and chronic illness.

#### 3. Endometriosis was my story

The impact of the chronicity of pain and its consequences in the daily life of patients with endometriosis appears to be highly negative. Indeed, pain and exhaustion related to the chronicity of the condition are altering the well-being of many respondents.

“*I realized two years ago that I am suffering of endometriosis and yes in fact it poisons my life … it is a process like a cancer but it’s not cancer… so good for me*, *I am lucky… I would not incriminate my burn-out to endometriosis only… but no doubt it played a role in terms of an exhausting condition*”. (Respondent 2).

Pain is perceived as chronic and experienced as having heavy physical and emotional negative consequences in most respondents. Eight out of ten respondents explain how endometriosis is not only a chronic but also a lifelong insidious condition with consequences on intimate and affective life:

“*I knew it is a chronic condition*. *I experience pain everyday but I am able to control it better with curcuma*, *ginger*, *and essential oils*. *But for the last three months I have been having pain during sexual intercourse which I can also control by changing positions*. *My worry now is after sexual act*, *pain is just terrible*. *During 20–30 minutes*, *I cannot unfold my body because of the pain*, *I cannot neither move nor get up* “(Respondent 8).

“*The illness is relatively silent*, *unless we have daily pain… like during periods or during sexual intercourse where it is triggering inside sensibility and emotions because we cannot have sex normally because of the pain*. *It is very difficult as intimacy and private life take a hit*”. (Respondent 10).

The emotional and physical burden are frequently expressed in the narratives with the figure of an illness that is “poisoning” most part of the women’s lives and their perception of being feminine. In the narratives, six respondents out of ten expressed how much endometriosis has a negative impact on many dimensions of their personal life. Women are struggling to fulfill their feminine identity despite chronic pain and to protect the couple’s intimacy despite disturbances on their sexuality, and also on fertility. “*After the diagnosis was made*, *I was an emergency case because of intestinal lesions…my pain was really reaching huge thresholds*. *They did two surgeries*, *pulmonary talcage and two weeks later another surgery […] Indeed the big big surgery*. *…It explains my* *lived* *experience with pain*, *it wasn’t any longer a fatality linked to being a woman*. *…I think I had a lot of anger*, *feelings of injustice*, *a lot of sadness… Endometriosis was my story*, *was my illness*. *[…] And infertility became a couple story… With a bit of hope*, *however*, *because infertility treatment existed in 2014* “(Respondent 7).

#### 4. And infertility became a couple story

The fear of being infertile is strongly expressed in most narratives. Medical procedures exist to support women and couples facing infertility. Techniques such as oocytes cryopreservation and the tendency to anticipate future fertility issues for young women are part of specialist consultations to increase the chance of fertility later in life.

These nineteen-year-old respondent narrates: “*My doctor advised to freeze my oocytes but it is a bit complicated with my parents at home… When I discussed the subject*, *it was…yes… but no… I don’t know what to do*, *how to do it*. *…I know endometriosis can damage the uterus and make welcoming a baby very problematical*, *with high risk of miscarriages… Ovaries may also be damaged so we cannot create good oocytes*.*”* (Respondent 1). This case underlines the importance of the patient-doctor relationship and the misunderstanding that can stay unspoken. Indeed, the specific therapeutic options brought up by the specialists for preventing a risk of probable infertility appears as disruptive for the 19 years-old respondent: it seems quite early for her to think about oocyte cryopreservation and the maternity issues as her transition to adulthood is still ongoing.

Suffering from endometriosis makes the project of having children a lot more complicated: fertility and fecundity are impaired, and this raises strong emotional issues: “*Endometriosis is always a source of anxiety and it takes more space in my mind with the passing of time because I am associating endometriosis with my difficulties becoming pregnant and having children if ever possible…It is also exhausting because the intimacy of the couple is troubled with that issue”* (Respondent 10).

Facing infertility represented an enormous struggle for most women. Respondent 3 reports: “my fear that it will last for ten years and that in the end it will not work”; moreover, as Respondent 5 stressed, “the desire to become pregnant became obsessive”.

In addition to their own struggle, women also face the challenge of dealing with infertility as a couple. Seven respondents out of eleven expressed that endometriosis created tensions and conflicts in the relationship. As dyspareunia can be exacerbated when contraception is discontinued for fertility reasons, sexuality may become problematic.

Involvement and support of the partner is not always available. For men, facing the distress and helplessness regarding the medical condition of their partner can also be a major source of anxiety. Furthermore, endometriosis may end up not being manageable in the relationship and lead to a break-up: “*In the end*, *endometriosis ruined our marriage*, *the illness had too much of an impact on him… and I’m now alone with no child”* (Respondent 9).

## Discussion and conclusion

The present study clearly points to the physical and psychological suffering related to endometriosis and its intricate consequences. In accordance with the results of a systematic review [35], our analysis of interviews corroborates the distressing impact of the trivialization of pain and the uncertainty of and the long quest for diagnosis. The findings also highlight several associated issues, from the diagnostic delay to the low success rates of fertility treatments. Our findings also stress the negative perceptions of initial medical care in which pain is frequently ignored and repeated complains remain unheard.

This qualitative analysis contributes to better understand the accumulation of negative emotions throughout the long illness-trajectory of endometriosis and the poor dyadic adjustment within couples. Moreover, women’s narratives show that when the diagnosis of endometriosis is reached after years of unexplained symptoms and diagnosis wandering, women experience a sense of reassurance, regain of control, and show better adjustment to the condition. This double-edged experience with healthcare professionals is well described in a qualitative study by Grundström et al. [36].

In their illness-trajectory, women describe their struggle on two battlefields: 1) fulfilling their feminine identity despite of chronic pain, 2) protecting the couple’s intimacy and sexuality. In the narratives, endometriosis is perceived as an insidious distressful process that grows from an individual emotional and physical suffering to a disruptor of the equilibrium within the relationship. Much in line with previous studies, our respondents also emphasize the threatening effects of this chronic condition on their lives as well as on those of their partners [19, 37–39].

The words used in the narratives illustrate the extent and the seriousness of the condition: indeed, these words echo with the idea of physical threat, handicap, fear of surgery and its possible mutilating consequences, but also with the threat to femininity and body-image integrity. In this regard, the mention of artificial menopause as a treatment of pain belongs to the same negative perception as it puts a threat not only on femininity but also on fertility.

Our results are consistent with those of a recent review of qualitative research that presents the perspective of 229 women, 61 men and 45 couples concerning endometriosis-related infertility. This review highlights the impact of endometriosis-related infertility on social and personal identity of being a woman, the impact of endometriosis on family planning, the revision of expected life trajectories in case of infertility treatment failures and the alteration of dynamics within the couple unit [40]. A recent qualitative study with in-depth interviews also showed how women’s experience is profoundly shaped by endometriosis-pain. Medical egg freezing was described as a disease-management strategy to cope with the potential future infertility and was perceived as a psychologically costly “additional” medical intervention [41].

The exploration of illness representations within a medical consultation allows the bodily experience, emotions, thoughts and traumatic load to be understood by the physician, legitimated and integrated in the therapeutic approach. Through empathic listening, women can verbalize the complexity of the lived experience and progressively develop a more coherent mental functioning. From a psychosomatic perspective the illness perception and lived experiences are unveiled during the narration and lead to a therapeutic alliance with the medical practitioner [15].

The qualitative study of Young et al. [7] discussed the potential conflicts between women and medical professionals when subjective priorities are not considered. This is the case in the example discussed above regarding the 19 years-old respondent who was proposed to “*freeze [her] oocytes”*, with her gynecologist privileging future fertility and hence oocytes vitrification over other aspects of care. Each complaint is singular and investigating its deeper meaning allows to collect its various dimensions such as ideas of illness-related identity, feminine affirmation, sexuality, fertility and maternity. In turn, addressing and sharing these subjective dimensions with the caregiver tend to open a narrative co-construction newly perceived as less traumatic and thus alleviating some of the illness-burden [7].

This study has both strengths and limitations. The triangulation of data analysis and interpretation is among the strengths. This analysis that was carried out by a multidisciplinary research team (psychiatrists, psychologist, reproductive endocrinologist, gynecologist) so that findings were confirmed through a consensus between the investigators, thus contributing to the credibility and validity of the findings.

However, as also pointed out in other studies [42], the women who volunteered to participate appeared to have gone through a lengthy process of reflecting on their condition and the course of possible treatment options, and the findings may thus not be applicable to all the women suffering from endometriosis.

Our study was carried out in a sample of 11 French-speaking women in Switzerland who participated in an online survey reproductive awareness and choices in women with endometriosis [13]. Our findings are therefore limited to that population and by the small sample size. Furthermore, partners and husbands were not interviewed, and we therefore only have the women’s perception of the impact of endometriosis on the couple and fertility [37]. As Hudson et al. suggested, separate interviews with dyadic analysis could lead to a better understanding of the complexity of infertility issue-related to endometriosis [43].

These results of this study have implications for clinical practice and give us some insight on how to approach fertility issues in women with endometriosis. Women’s narratives highlight that fertility issues cannot be isolated from the lived experience and the complexity of endometriosis-related repercussions on the individual and on the couple. Furthermore, our findings show the complicated relationship between women suffering from endometriosis and healthcare providers. Indeed, women’s stories report pain trivialization, lack of interest and medical knowledge as well as lack of information provision and medical wandering without diagnosis or insufficient treatment provision. Our findings corroborate the findings of Ng et al. that showed a negative perception of the medical profession that was associated with surgical treatment failure, emergency room use for pain management and the use of complementary health care [39].

Endometriosis is a condition that affects women throughout their reproductive life span from menarche to menopause and current therapeutic approaches aim at relieving pain, maintaining quality of life, preserving fertility, or treating infertility, and reducing the physical, sexual, social and psychological negative consequences of the disease.

Although time consuming, a patient-centered type of investigation allows disclosure of patient’s illness perceptions and representations and the understanding on how the multiple repercussions of endometriosis impact fertility and the project to have a child. Exploring patient’s illness perceptions involves a training in communication skills and learning how to access the world of patients’ representations and perceptions [16]. Recently, several tools to assess the multidimensional burden of endometriosis and the long-term impact on different aspects of women’s lives have been developed and could be integrated in endometriosis care [44, 45]. Further studies should assess how gynecologists can better apprehend the complex subjective dimensions that contribute to the illness perceptions and integrate them in the therapeutic approach.

## Supporting information

S1 Checklist Consolidated criteria for reporting qualitative studies (COREQ): 32-item checklist. (DOCX)
