# Correction: Women’s lived experience of endometriosis-related fertility issues

**DOI:** 10.1371/journal.pone.0312142

**Published:** 2024-10-10

**Authors:** The PLOS ONE Editors

There is an error in the Methods reporting of ethical approval for the study. The correct ethics statement is:—“The authors took care to conform to the ethical standards promoted in the Declaration of Helsinki. The study protocol was approved by the ethics committee of the canton of Geneva, Switzerland and all participants signed an informed consent form.”

The publisher apologizes for the error.
