# Supplementary material for: Women’s lived experience of endometriosis-related fertility issues
Source: PLoS One. 2023 Nov 6;18(11):e0293531. doi: 10.1371/journal.pone.0293531 (PMC10627452; doi:10.1371/journal.pone.0293531)
Supplement: S1 Checklist — (DOCX) [file pone.0293531.s001.docx]

**Table COREQ**

**Consolidated criteria for reporting qualitative studies (COREQ): 32-item checklist**

| **No** | **Item** | **Guide questions/description** |
| --- | --- | --- |
| **Domain 1: Research team and reflexivity** |  |  |
| Personal Characteristics |  |  |
| 1. | Interviewer/facilitator | First and second authors |
| 2. | Credentials | Both are MDs, with a certification in psychiatry |
| 3. | Occupation | Both involved in liaison-psychiatry at the maternity of the Geneva University Hospitals |
| 4. | Gender | Both female |
| 5. | Experience and training | Both trained in qualitative interview methods and certified psychotherapists |
| Relationship with participants |  |  |
| 6. | Relationship established | None with the participants prior to the study |
| 7. | Participant knowledge of the interviewer | No personal information |
| 8. | Interviewer characteristics | Both had been working in liaison-psychiatry and women’s health for a number of years |
| **Domain 2: study design** |  |  |
| Theoretical framework |  |  |
| 9. | Methodological orientation and Theory | Grounded theory; content analysis |
| Participant selection |  |  |
| 10. | Sampling | Purposive and consecutive*,* proposed to women who had participated in a first quantitative study as described in the manuscript |
| 11. | Method of approach | Contact by telephone and face-to-face interviews |
| 12. | Sample size | 11 |
| 13. | Non-participation | 1 dropped-out dropped: no desire of maternity and no infertility problems |
| Setting |  |  |
| 14. | Setting of data collection | Infertility clinic at the Geneva university Hospitals |
| 15. | Presence of non-participants | No |
| 16. | Description of sample | Age ranged from 19 years to 34-years-old, professions were very diverse. See Table 2 |
| Data collection |  |  |
| 17. | Interview guide | See Table 1. The interview was pilot tested on two patients. |
| 18. | Repeat interviews | No |
| 19. | Audio/visual recording | Audiorecording was used to collect the data |
| 20. | Field notes | Yes |
| 21. | Duration | Interviews lasted 45–75 minutes |
| 22. | Data saturation | Yes, see page 5 of the manuscript |
| 23. | Transcripts returned | No |
| **Domain 3: analysis and findings**z |  |  |
| Data analysis |  |  |
| 24. | Number of data coders | Three in a first round, then discussed with the whole multidisciplinary team |
| 25. | Description of the coding tree | No |
| 26. | Derivation of themes | Themes were derived from the data |
| 27. | Software | Not applicable |
| 28. | Participant checking | No |
| Reporting |  |  |
| 29. | Quotations presented | Participant quotations are presented to illustrate the themes / findings, along with an identification for each quotation. |
| 30. | Data and findings consistent | Yes, see results and discussion sections. |
| 31. | Clarity of major themes | Yes |
| 32. | Clarity of minor themes | Major themes are presented first and secondary themes in a second intention. |
